# Characterization of lactate utilization and its implication on the physiology of *Haemophilus influenzae*

**DOI:** 10.1016/j.ijmm.2014.02.010

**Published:** 2014-05

**Authors:** Sabine Lichtenegger, Isabelle Bina, Sandro Roier, Stilla Bauernfeind, Kristina Keidel, Stefan Schild, Mark Anthony, Joachim Reidl

**Affiliations:** a Institute of Molecular Biosciences, University of Graz, Humboldtstr. 50, 8010 Graz, Austria; b Insitute of Hygiene and Microbiology, University of Wuerzburg, Josef-Schneider Str. 2 E1, Wuerzburg 97080, Germany; c Department of Paediatrics, Oxford University Hospitals, Headington, Oxford OX3 9DU, United Kingdom

**Keywords:** l-Lactate utilization, *lctP*, *Haemophilus influenzae*, Colonization

## Abstract

*Haemophilus influenzae* is a Gram-negative bacillus and a frequent commensal of the human nasopharynx. Earlier work demonstrated that in *H. influenzae* type b, l-lactate metabolism is associated with serum resistance and in vivo survival of the organism. To further gain insight into lactate utilization of the non-typeable (NTHi) isolate 2019 and laboratory prototype strain Rd KW20, deletion mutants of the l-lactate dehydrogenase (*lctD*) and permease (*lctP*) were generated and characterized. It is shown, that the apparent *K*_M_ of l-lactate uptake is 20.1 μM as determined for strain Rd KW20. Comparison of the COPD isolate NTHi 2019-R with the corresponding *lctP* knockout strain for survival in human serum revealed no lactate dependent serum resistance. In contrast, we observed a 4-fold attenuation of the mutant strain in a murine model of nasopharyngeal colonization. Characterization of *lctP* transcriptional control shows that the lactate utilization system in *H. influenzae* is not an inductor inducible system. Rather negative feedback regulation was observed in the presence of l-lactate and this is dependent on the ArcAB regulatory system. Additionally, for 2019 it was found that lactate may have signaling function leading to increased cell growth in late log phase under conditions where no l-lactate is metabolized. This effect seems to be ArcA independent and was not observed in strain Rd KW20. We conclude that l-lactate is an important carbon-source and may act as host specific signal substrate which fine tunes the globally acting ArcAB regulon and may additionally affect a yet unknown signaling system and thus may contribute to enhanced in vivo survival.

## Introduction

The only known natural habitat of *Haemophilus influenzae* is the human nasopharynx. It thus experiences a relatively constant and stable environment, and has retained a certain degree of metabolic flexibility in the form of a limited metabolic and substrate utilization pathway (Edwards and Palsson, 1999). This limited flexibility may allow adaptation in the face of nutrient restriction, or adaptation to varying microenvironments within the nasopharynx. A carbon source, which is available in the human body, circulating in serum, mucosa, and saliva is l-lactate, found in concentrations of 0.3–1.3 mM (Lentner et al., 1981). l-Lactate is also available in the nasopharynx of mice (Exley et al., 2005a) and is additionally supplemented as sodium l-Lactate in the defined growth medium MIc (Herriott et al., 1970).

In *Escherichia coli*, utilization of l- and d-lactate enantiomers is accomplished by LctP and the glycolate transporter, GlcA (Núñez et al., 2001). Both permeases can transport d- and l-lactate, and glycolate. The *lct* gene cluster of *E. coli* is organized in an operon consisting of *lctPRD*, whereby *lctR* encodes a transcriptional factor and *lctD* encodes for flavin-linked l-lactate dehydrogenase (Dong et al., 1993). Under aerobic conditions, *lctP* and *lctD* genes are induced in the presence of l-lactate, but not d-lactate. The expression of *lctD* and other genes is elevated under aerobic and repressed under anaerobic conditions (Iuchi et al., 1994). This regulation is mediated by the two-component anoxic redox control system ArcAB, whereby under anaerobic conditions the response regulator ArcA is phosphorylated by the sensor kinase ArcB (Malpica et al., 2004) and acts as a repressor for *lctD* gene expression (Iuchi et al., 1994).

Not much is known about lactate metabolism in *H. influenzae*. An earlier report describes a stimulatory effect on the development of competence by the addition of l-lactate to competence inducing growth medium (Miller and Huang, 1972). Another report shows that the addition of glucose and lactate enhances the cell mass of growing *H. influenzae* strains, indicating that lactate serves as carbon-source (Hollander, 1976). In 1990, the d-lactate dehydrogenase of *H. influenzae* was characterized (Denicola-Seoane and Anderson, 1990). Finally, it was shown that under hemin limiting growth conditions and glucose derived growth, d-lactate accumulates as an end product in an NTHi strain, indicating high d-lactate dehydrogenase activity in vivo (O‘Reilly et al., 1992).

*H. influenzae* type b (Hib) is able to develop resistance to the bactericidal activity of blood serum (Shaw et al., 1976). Interestingly, an association between lactate metabolism and virulence attributes was published by Kuratana and Anderson in 1991, showing an increase of serum resistance of Hib in the presence of l-lactate (Kuratana and Anderson, 1991). Two mechanisms of serum resistance were proposed (Kuratana et al., 1990). In the first, bacteria pre-incubated with a buffer containing glucose, lactate, urea and bicarbonate develop serum resistance together with an observed quantitative increase in lipooligosaccharides (LOS) and capsular polysaccharides. In the second, a mixture of lactate plus calcium led to serum resistance only associated with an increase in capsular polysaccharides (Kuratana et al., 1990, Kuratana and Anderson, 1991, Smith, 2000). Recently, in encapsulated as well as non-typeable *H. influenzae* (NTHi) strains, changes in LOS structures were shown to be responsible for alterations in serum resistance (for overview see Hallström and Riesbeck, 2010). Interestingly, Wong et al. reported that ArcA regulates a LOS specific glycosyltransferase, encoded by *lic2B*, which affects serum resistance in one clinical NTHi isolate (Wong et al., 2011). A similar phenotype was also identified earlier, by testing survival of an Hib *arcA* mutant strain in human serum (De Souza-Hart et al., 2003). However, in Hib strains the responsible ArcA regulated and serum survival-associated-target genes were not identified.

As known for *Neisseria meningitidis*, l-lactate is a substrate for the synthesis of N-acetyl-neuraminic acids via the NeuB synthetic enzyme and thus contributes to enhanced serum resistance (for overview see Smith et al., 2007, Vimr et al., 2004). To prove a supporting role of lactate in serum resistance, *N. meningitidis lctP* mutants were characterized. It was shown that a loss of the ability to use l-lactate correlates with a loss of serum resistance (Exley et al., 2005b). Additionally, the inability to metabolize lactate led to attenuated nasopharyngeal colonization (Exley et al., 2005a), growth in the bloodstream and cerebrospinal fluids in an animal model (Exley et al., 2005b). Further analysis additionally revealed an attenuation of an *N. gonorrhoeae lctP* mutant in the murine model of lower genital tract infection (Exley et al., 2007), leading to the overall observation that *Neisseria* use host lactate sources (Smith et al., 2007). In contrast, l-lactate is not a substrate for the synthesis of N-acetyl-neuraminic acids in *H. influenzae*. Instead exogenous N-acetyl-neuraminic acids are utilized via the SiaPQM uptake system to become a substrate for Lic3A, SiaA and LsgB, or to be metabolized (for review see, Vimr et al., 2004). Recent studies, investigating NTHi as well as Rd strains, revealed that incorporation of sialic acids into LOS and changes in outer membrane compositions hinder and prevent antibody recognition of conserved parts related to LOS and thus enhance serum resistance (for overview see, Clark et al., 2013, Hallström and Riesbeck, 2010, Nakamura et al., 2011). Genetically, phase variable LOS biosynthetic genes, such as *lic3A* (Hood et al., 2001), *lex2A* and *lgtC* (Clark et al., 2013), contribute to and enhance the incorporation of compounds such as choline or neuraminic acids into the LOS structure.

As emphasized earlier, previous work (Kuratana et al., 1990, Kuratana and Anderson, 1991) reported enhanced serum resistance in response to the presence of lactate in Hib. Thereby, l-lactate may act as a host factor influencing the virulence behavior of *H. influenzae* (Smith, 2000). Little is known about *H. influenzae* lactate metabolism during in vivo colonization of the nasopharynx. Signature tagged mutagenesis in Rd-b^+^ revealed a transposon insertion in the l-lactate permease encoding gene *lctP* which led to decreased in vivo survival in a blood stream infection model of infant rats (Herbert et al., 2002). Of high interest is the observation that a connection seems to exist between the ArcAB regulon and lactate metabolism. Similarly to *E. coli* (Iuchi et al., 1994), transcription of the *H. influenzae* lactate permease and l-lactate dehydrogenase encoding genes is under the control of ArcA (Wong et al., 2007). To further assess l-lactate utilization and its contribution to the development of serum resistance and colonization in the nasopharyngeal region we created defined deletion mutants in *arcA* and the l-lactate pathway, i.e. permease (*lctP*; HI1218), l-lactate dehydrogenase (*lctD*; HI1739.1), and characterized their influence on bacterial physiology.

## Materials and methods

### Bacterial strains, culture conditions and growth analysis

Strains used in this study were Rd KW20 (gift of A. Wright, Tuffs University, Boston), which is an unencapsulated former type d strain, and NTHi COPD isolate 2019-R (Roier et al., 2012) (original strain was a gift of M. Apicella, University of Iowa, Iowa City). For mutant strain constructions, *E. coli* strain MC4100 (Casadaban, 1976) or LE392 (Maniatis et al., 1989) were used. *H. influenzae* wild type or mutant strains were cultured either in BHI broth supplemented with NAD (10 μg/ml) and hemin (20 μg/ml) or Mlc medium (Herriott et al., 1970). l-Lactate (7 mM) was added as indicated. Kanamycin (Km 10 μg/ml), streptomycin (Sm, 100 μg/ml) and chloramphenicol (Cm, 2 μg/ml) were added where appropriate. *Escherichia coli* strains were cultured in Luria-Bertani (LB) broth or on LB agar plates, supplemented where necessary with Km (50 μg/ml) or Cm (10 μg/ml). All supplements were purchased from Sigma.

### Ethical statement

BALB/c mice (Charles River) were used for competitive colonization experiments in strict accordance with the Guide for the Care and Use of Laboratory Animals of the National Institutes of Health, the national “Bundesgesetzblatt fuer die Republik Oesterreich”. Animal protocol (39/158 ex 2000/10) has been approved by the Austrian Federal Ministry of Science and Research Ref. II/10b and the Committee on the Ethics of Animal Experiments of the University of Graz. Housing of mice was conducted with food and water ad libitum and monitored in accordance with the rules of the Institute of Molecular Biosciences at the University of Graz.

### Deletion and insertion mutagenesis of *lctP*, *lctD* and *arcA* and *lctP* complementation

Chromosomal and plasmid DNA were extracted using the method described by Grimberg et al. (Grimberg et al., 1989) and spin-column technology (Qiagen), respectively. PCRs for sequencing and subcloning were carried out using the Phusion™ High-Fidelity polymerase (NEB). For all other reactions, Taq DNA Polymerase (NEB) was used. For generating *lctP*::, *lctD*:: and *arcA*::*cat* knockout mutants, about 500 bp upstream and downstream respectively to the start and stop codons were targeted. DNA fragments were produced by PCR, using oligos (Table 1) for *lctP* upstream: lctPA-5′-KpnI and LctPA-3′-XhoI. For *lctP* downstream: lctPE-5′-BamHI and lctPE-3′-SacII. For *lctD* upstream: lctDA-5′-PstI and lctDA-3′-BamHI. For *lctD* downstream: lctDE-5′-XbaI and lctDE-3′-PstI. For *arcA* upstream: arcAA_PstI_5′-3′ and arcAA-BamHI-3′-5′. For *arcA* downstream: arcAE-XbaI-5′-3′ and arcAE-PstI-3′-5′. Oligos (Table 1) for the PCR amplification of the *cat* gene cassette of pACYC184 (Rose, 1988b), used to replace *lctD* and *arcA*, were utilized as follows: cat-5′-BamHI and cat-3′-XbaI. Oligos for the PCR amplification of *cat* gene cassette of pACYC184 for replacement of *lctP* were used as follows: cat-5′-XhoI and cat-3′-BamHI. For the knockout constructions *lctD*::*cat* and *arcA*::*cat*, upstream and downstream PCR fragments were ligated with the amplified fragment of the chloramphenicol resistance encoding gene cat and subcloned into PstI digested plasmid pSK BluescriptII (Stratagene). Subsequently after ligation and transformation these plasmids were prepared and cut with PstI. The obtained PstI DNA fragments were then used for transformation into hypercompetent strain sxy-1 (*tfoX*^*c*^ Rd KW20) (Redfield, 1991). In parallel, *lctP*::*cat* ligation products were amplified by PCR using oligos lctPA-5′-KpnI and lctPE-3′-SacII and the resulting PCR fragment was also transformed into *H. influenzae* strain sxy-1. After plating chloramphenicol resistant (Cm^R^) sxy-1 transformants were isolated and using PCR technique, correct *cat* insertion and replacement of *lctP*, *lctD* and *arcA* genes was verified (data not shown). For transformation of genetic constructions into wild type strains Rd KW20 and 2019-R, cells were grown to mid-logarithmic phase in BHI medium, as it is was shown that *H. influenzae* becomes naturally competent under these conditions (Maughan and Redfield, 2009, Redfield et al., 2006). Additionally, to construct an *lctP arcA* double mutant the *cat* cassette of the *arcA* construct was exchanged by a kanamycin resistance gene. This gene was obtained from plasmid pACYC177 (Rose, 1988a), using the oligonucleotides kan-5′-BamHI and kan-3′-XbaI. The residual cloning strategy matches the above described procedure. First, *arcA* was exchanged by the kanamycin resistance gene in strain Rd KW20. Using isolated chromosomal DNA from this mutant strain, a PCR fragment was generated using the oligonucleotides ArcA_fw and ArcA_rv. The obtained fragment was then transferred into the *lctP* single mutant to yield the *lctP arcA* double mutant (Km^R^ Cm^R^). For Rd Δ*p5* and 2019-R Δ*p5* an approach, similar to the already described mutagenesis, was applied. The oligonucleotides P5_up fw1 [EcoRI]/P5_up rv2 [PstI][SnaBI] and P5_down fw3 [PstI]/P5_down rv4 [HindIII] were used to generate an about 800 bp fragment upstream and downstream of *p5*. For the amplification of the fragments the particular chromosomal DNA was used (2019-R or Rd KW20). The two fragments, separated by a gene conferring Cm^R^, were ligated into the vector pUC19 (Yanisch-Perron et al., 1985). The Cm^R^ gene originated from a digestion of pAKcat (Kraiß et al., 1998) by SnaBI and PstI. Following ligation, the plasmid was transformed into DH5αλpir (Hanahan, 1983) for amplification. The by PCR verified and re-isolated plasmid served as a template to generate a PCR fragment consisting of the upstream and downstream fragment as well as the *cat* cassette. This fragment, isogenic to the 2019-R DNA, was then transformed into the 2019-R strain and positive *p5* mutants were verified by a PCR reaction (data not shown). The Rd KW20 fragment was first transformed into the sxy-1 strain. After the selection of *p5* mutants the chromosomal DNA was re-isolated and transformed into the Rd KW20 strain, which was afterwards again checked for the *p5* deletion by PCR (data not shown).

**Table 1:** Oligonucleotides used in this study.

Oligo name	5′-3′
ArcA_fw	TATCTGTAGATAAAGTAGCAGAAAA
ArcA_rv	TAATTTCCACACTTACAATAGATTG
arcAA_PstI_5′-3′	ATACTGCAGATTTACCGACTAACTCGCACT
arcAA-BamHI-3′-5′	ATAGGATCCATTTGCCAATATATGATGCATTT
arcAE-XbaI-5′-3′	ATATCTAGAACAATTCGACGTATCAGAAAACA
arcAE-PstI-3′-5′	ATACTGCAGATTGACTCAATTGTCATTGTGAT
cat-5′-BamHI	CCGAGGATCCGTAAGTTGGCAGCATCACCCG
cat-3′-XbaI	CCGATCTAGAAGGCGTTTAAGGGCACCAATAA
cat-5′-XhoI	CCGACTCGAGGTAAGTTGGCACATCACCCG
cat-3′-BamHI	CCGAGGATCCAGGCGTTTAAGGCACCAATAA
kan-5′-BamHI	TAAGGATCCTTCAACTCAGCAAAAGTTCGA
kan-3′-XbaI	TAATCTAGATCAGCGTAATGCTCTGCCA
lctDA-5′-PstI	TTGACTGCAGGAGTTATCTTCGTGGGCAGG
lctDA-3′-BamHI	TCAAGGATCCACATAAAGGGAGGAACACGGC
lctDE-5′-XbaI	TTCATCTAGAGCCGATATTAAACCAGAGG
lctDE-3′-PstI	ATGACTGCAGATTAGCGACAGTTTCCCGTC
lctPA-5′-KpnI	CCGAGGTACCGTGAGAATTTGTAATCCTGC
LctPA-3′-XhoI	CCGACTCGAGCGTAATAGGTGTTGTACCGC
lctPE-5′-BamHI	CCGATGGATCCCGGTATCATTGCCGCACTTG
lctPE-3′-SacII	CCGATCCGCGGCCAGGATACTTCGAATCAC
lctPx_fw	TGAACAAACAGTTAAATCGGAAAC
lctP.kan.rv_up.rv	TCTTGTGCAATGTAACATCAGAGTTTAATGAAAATACCTAAAAAATAATAAA
lctP.up.fw_kan.fw	TTTATTATTTTTTAGGTATTTTCATTAAACTCTGATGTTACATTGCACAAGA
lctP.kan.fw_down.fw	GTCAGCAACACCTTCTTCACGTTTATTAAATCATTCCTTGAGATCC
lctP.down.rv_kan.rv	GGATCTCAAGGAATGATTTAATAAACGTGAAGAAGGTGTTGCTGAC
lctPy_rv	ATACGGTAATCTTGAGATACTTCA
P5_up fw1 [EcoRI]	TAAGAATTCGAGAAAGATCCACTATTATATTGTT
P5_up rv2 [PstI][SnaBI]	TAACTGCAGTAATACGTATTTGATGTCCTCTATTTAGTGATC
P5_down fw3 [PstI]	TAACTGCAGAACGAAAGATTAAATACAGCAAAAG
P5_down rv4 [HindIII]	TAAAAGCTTCAAACAACCTGCCGCACCAAT
RT-rpoA_fw	AGGAAGGTGTTCAAGAAG
RT-rpoA_rv	GAGATGAAGCAGGAACATA
RT-lctP fw	GCGCTACCTTCTTATGTT
RT-lctP rv	AAACGCCCAACCAATAA

For the purpose of complementation, the *lctP* gene was chromosomally restored in an *lctP* mutant. Therefore an overlap extension PCR was performed as described earlier (Horton et al., 1989). Briefly, three fragments were amplified with two originating from the Rd KW20 wild type chromosome. One fragment contains a region upstream of the *lctP* gene as well as the gene itself, comprising an overall size of about 2740 bp, and is designated the “upstream fragment”. The second fragment, which was amplified from the Rd KW20 chromosome, consists of about 760 bp downstream of the *lctP* gene and is designated the “downstream fragment”. Oligonucleotides lctPx_fw/lctP.kan.rv_up.rv and lctP.kan.fw_down.fw/lctPy_rv were used to yield the upstream and downstream fragment, respectively. The third amplicon confers kanamycin resistance and was amplified from the plasmid pUC4kan (Vieira and Messing, 1982), using oligonucleotides lctP.up.fw_kan.fw and lctP.down.rv_kan.rv. Both, the upstream and downstream fragments further contain overlapping regions, corresponding to the 5′ and 3′ end of the kanamycin gene. These overlapping regions allow the fragments to anneal in a further PCR reaction step, which in the end leads to a 4.6 kb fragment. The final product consists of the upstream region, the *lctP* gene (2.7 kb) and the downstream region (0.7 kb) with the inserted kanamycin resistance cassette (1.2 kb). Bacteria were then mixed with the PCR fragment and after an hour incubation step, they were plated on kanamycin plates. This allows the selection of strains now harboring the Km^R^ on their chromosome. Nevertheless, some of the selected transformants could also harbor the *lctP::cm* insertion gene, as the kanamycin cassette and *lctP* are integrated via homologous recombination of the upstream and downstream region with the corresponding regions on the chromosome. To further evaluate the chromosomal restoration strains were tested for chloramphenicol sensitivity, as the Cm^R^ should be lost in the process in some transformants. Such transformants were identified by Cm^S^ and Km^R^ and were subsequently characterized by PCR using *lctP* flanking oligos to obtain a normal size *lctP* gene product (data not shown). All strains used and generated in this study are listed in Table 2.

**Table 2:** Strains used in this study.

Strain	Description	Reference
***E. coli*****strains**		
MC4100	F− *araD139* (*argF-lac*)*U196 rpsL150* (St^r^) *relA1 flbB5301 deoC1 ptsF25 rbsR*	(Casadaban, 1976)
LE392	F− *supF supE hsdR galK trpR metB lacY tonA*	(Maniatis et al., 1989)
DH5αλpir	F− *endA1 glnV44 thi-1 recA1 relA1 gyrA96 deoR nupG* Φ80d*lacZ*_M15_(*lacZYAargF*) U169, *hsdR17*(rK− mK+), λpirRK6	(Hanahan, 1983)
***H. influenzae*****strains**		
Rd KW20	unencapsulated variant of a former type d strain	A. Wright
Rd Δ*lctP*	*lctP*	This study
Rd Δ*arcA*	*arcA*	This study
Rd Δ*lctD*	*lctD*	This study
Rd Δ*p5*	*p5*	This study
2019-R	clinical NTHi isolate from a patient with chronic obstructive pulmonary disease	(Campagnari et al., 1987)
2019-R Δ*lctP*	*lctP*	This study
2019-R Δ*arcA*	*arcA*	This study
2019-R Δ*lctP* Δ*arcA*	*lctP, arcA*	This study
2019-R Δ*p5*	*p5*	This study

### Preparation of RNA and qRT PCR

To determine *lctP* expression levels in various strain backgrounds, six independent cultures were grown to early exponential phase. Upon requirement cultures were supplemented with l-lactate (7 mM). The extraction of bacterial RNA was carried out using the RNeasy Mini Kit (Qiagen) and residual chromosomal DNA was removed applying RQ1 RNAse-Free DNase (Promega) according to the manufacturer’ protocol. The synthesis of cDNA was accomplished using 200 ng bacterial RNA and the iSript Select cDNA Synthesis Kit (Bio-Rad). For the quantitative RT-PCR reaction SYBR GreenER qPCR SuperMix for ABI PRISM instrument (Invitrogen) and StepOne^TR^ Plus Real Time PCR System (Applied Biosystem) were utilized according to the manufacturer’ description. The reaction mix further contained 10 ng template DNA and 200 nM qRT_PCR oligonucleotides, which are listed in Table 1. Oligonucleotides are termed x_fw and x_rv, x designating the respective gene. Reactions were carried out in triplicates. The mean cycle threshold of the investigated transcript was normalized to the housekeeping gene *rpoA*.

### Bactericidal assay

The bactericidal assay was performed as previously described (Jarvis, 1994) and modified as follows. Briefly, mid-log grown bacteria were diluted 1:10 in PBS buffer supplemented with CaCl_2_, MgCl_2_ and bovine serum albumin. Cultures were either grown aerobically in MIc medium or anaerobically in BHI medium, both supplemented with l-lactate. BHI medium was chosen for anaerobical growth, as MIc medium does not represent a suitable growth medium for strain 2019-R under these conditions. As serum source, NHS (normal human serum, 2% used for Rd KW20 and 3% used for 2019-R), obtained from 5 healthy volunteers (taken according to approval of University Ethic commission GZ. 39/31/63 ex 2012/13) was added to the bacterial suspension with a final reaction volume of 250 μl. Additionally, bacteria were incubated in buffer to calculate the percentage of surviving cells without serum activity and to normalize the results. The final mixture was incubated for 45 min at 37 °C. For the investigation of a potential 2019-R *lctP* attenuation we mixed the wild type and Δ*lctP* strain prior to incubation with serum. The ratio of viable wild type to *lctP*::*cat* cell numbers was determined after plating serial dilutions. To exclude a serum effect due to metabolic differences the same assay was performed using 3% of heat inactivated serum, which results from incubation at 56 °C for 30 min. To evaluate our serum resistance assay we investigated the currently identified serum sensitive mutant *p5* (Rosadini et al., 2013) in the two strain backgrounds, 2019-R and Rd KW20. In contrast to the above described assay we incubated the wild type and the *p5* mutant separately in NHS.

### Nasopharyngeal colonization experiments

Nasopharyngeal colonization experiments were done with slight modification to a previously described procedure by Roier et al. (2012). Briefly, cultures were grown to mid-logarithmic phase, washed and adjusted to an OD_490_ of 0.1 in PBS buffer. Prior to infection the wild type and *lctP* mutant strain were mixed in a 1:1 ratio. To determine the actual inoculum and wild type to mutant ratio serial dilutions were plated on Sm as well as on Sm/Cm BHI agar plates. Anesthetized mice were then infected intranasally with 10 μl of the bacterial mixture. As a control, the same infection mix was used to inoculate MIc medium, supplemented with l-lactate or BHI medium. After 24 h mice were sacrificed and the dissected nasopharynx was mechanically homogenized. As previously described for the input, the nasopharyngeal homogenate was plated on both Sm and Sm/Cm BHI agar plates to distinguish wild type from mutant strains. Additionally, the competition index for the MIc and BHI medium control was determined by the same procedure.

### l-Lactate uptake and kinetics

For substrate uptake and kinetics, labeled substrate was applied as follows, l-(3-^14^C)-lactic acid sodium salt with specific activity of 55 mCi/mmol (Hartmann Analytic). For uptake studies, wild type and *lctP* mutant strains of Rd KW20 and NTHi 2019-R were used. Further the Rd KW20 *lctP* complementation strain and the *lctD* and *arcA* mutant were tested in this assay. Usually, *H. influenzae* strains from cultures grown to OD_490 nm_ 0.5 in Mlc medium, were washed and resupended in Mlc medium devoid of l-lactate. Subsequently, cultures were aliquoted in 3 ml and incubated with l-(3-^14^C)-lactic acid mixed with l-lactate (1:8 ratio) at an end concentration of 40 μM. Accordingly to given time tables, samples of 500 μl were removed at 20, 40, 60, 120 and 240 s (Fig. 3, Fig. 6). The samples were filtered through ME 25 filters (0.45 μm, Schleicher & Schuell, MicroScience) which membranes had been soaked with deionised water. The filters were washed with 10 ml PBS and placed in vials containing 3 ml of scintillation liquid (Ultima Gold™, PerkinElmer™). Radioactivity was measured in a scintillation counter (Tri-Carb 2300TR, Packard).

For substrate dependent uptake kinetics, the wild type strain Rd KW20 was used and cultures were prepared as previously described. The uptake reactions were started each with the addition of 20, 40, 50, 60, 80, 100 μM l(3-^14^C)-lactate. After 10, 30 and 50 s, samples of 500 μl were taken, filtered through membrane filters, washed under suction with 10 ml PBS and measured as previously described. To detect the entire added radioactivity per volume tested, 500 μl of the whole reaction mixture were added directly into vials containing 3 ml scintillation liquid. According to Xavier et al. (1996) transport rates derived from the counts per minute (cpm), obtained after 30 s of incubation with the labeled substrate, were determined. Thereby, it was tested whether a linear correlation of the number of counts versus time was observed for at least 30 s for all the substrate concentrations tested.

## Results and discussion

### Characterization of l-lactate utilization in *H. influenzae* for growth in complex medium

**Fig. 1:**
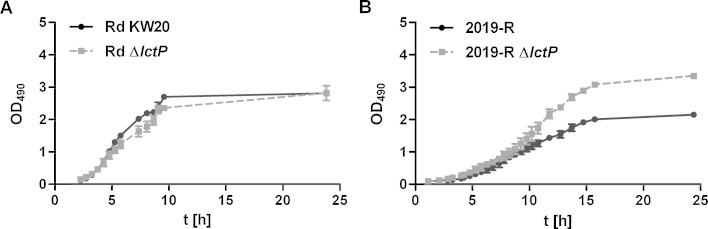
Growth of *H. influenzae* strains in Mlc medium supplemented with l-lactate. Shown are the growth curves of the wild type and the *lctP* mutant for both, strain Rd KW20 (A) and 2019-R (B). Data represent the mean values of three independent OD_490_ measurements as indicated by the SD error bars.

**Fig. 2:**
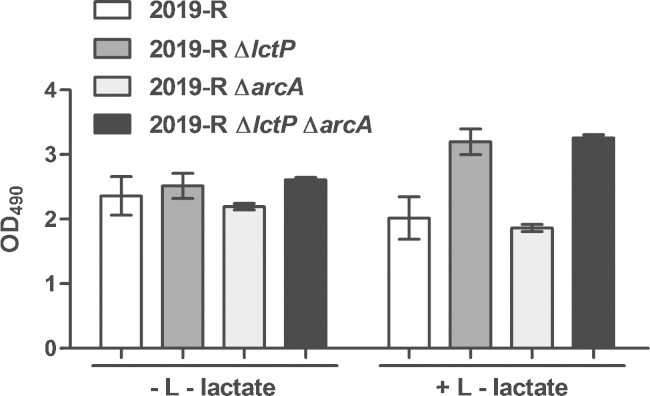
Optical density of growing bacteria. Shown are OD_490_ measurements of 2019-R wild type, *lctP*, *arcA* and *lctP arcA* mutants after 24 h of growth in Mlc medium without l-lactate (A) or supplemented with l-lactate (B). Bars represent the mean of at least three independent cultures. Error bars indicate the standard deviation.

To our knowledge no defined minimal growth medium, containing a single carbon source, exists for *H. influenzae*. However, sodium l-lactate (7 mM) is routinely added as supplement in a synthetic growth medium, termed Mlc (Herriott et al., 1970). In contrast no information about the lactate content of BHI medium is available. To test lactate utilization in *H. influenzae*, defined *lctP* (HI1218), *lctD* (HI1739.1) and *arcA* (HI0884) knockout mutants were generated in strain Rd KW20 replacing the genes by a kanamycin or chloramphenicol resistance gene cassette of plasmids pACYC177/184 (Rose, 1988a, Rose, 1988b) (see Materials and methods). Further constructions involved transfer of *lctP*::*cat* and *arcA*::*cat* mutations into the NTHi 2019-R background. To characterize growth, Rd KW20, NTHi 2019-R and corresponding *lctP* mutants were compared. For Rd KW20 and the *lctP* knockout strain, it was found that growth in Mlc showed no significant difference (Fig. 1A). In contrast, growth of NTHi strain 2019-R was altered in the mutant compared to the wild type (Fig. 1B). Interestingly, Δ*lctP* indicated faster growth starting in mid-log growth phase and reached higher optical densities in the stationary phase, e.g. after 24 h. To characterize whether this effect is due to the presence of l-lactate additional growth experiments, determining the optical density at 24 h, were performed. In fact the enhanced growth ability of the *lctP* mutant (Fig. 2), observed for the 24 h value, is dependent on the presence of l-lactate.

Why does an *lctP* mutant show enhanced growth ability in the late log and stationary phase when compared to the wild type strain? First to consider is that the observed result is strain dependent, since strain Rd KW20 is not showing such an effect. For strain 2019-R Δ*lctP*, it seems that presence of l-lactate may affect overall cell growth ability. To further characterize this phenomenon, we tested the behavior of the *arcA* knockout mutant as well as the *lctP arcA* double mutant. Interestingly, the results (Fig. 2) show no difference between wild type and *arcA* mutant growth abilities. Additionally, the double mutant displays the *lctP* phenotype. Because of the herein observed data we tend to exclude the ArcAB system to be responsible for the enhanced growth phenotype of the *lctP* mutant. Therefore, the question remains on how l-lactate influences the growth ability of the 2019-R isolate, which needs further characterizations.

### l-lactate uptake, complementation and transport kinetics

**Fig. 3:**
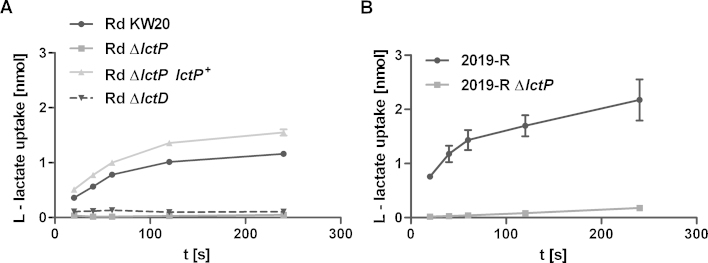
l-Lactate uptake by Rd KW20 (A) as well as 2019-R (B) wild type and *lctP* mutant strains. For Rd KW20 also the *lctP* complementation and the *lctD* mutant strain are included. The time dependent uptake was determined by using L-(3-^14^C)-lactate and is specified in nmol per 500 μl of culture volume (OD_490_ = 0.5). Shown are the mean values and SD error bars of at least two independent experiments.

**Fig. 4:**
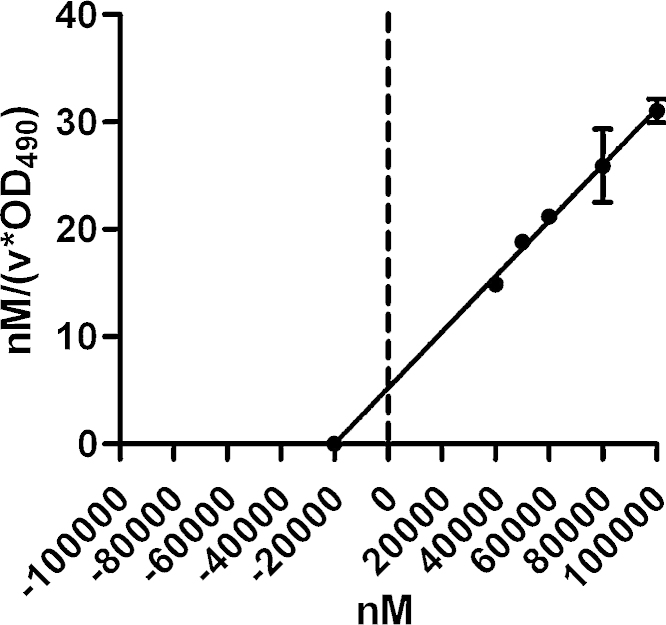
Hanes–Woolf Plot with *K*_M_ and *V*_max_ for l-lactate. The uptake velocities of different substrate concentrations were determined by measurements at three time points in strain Rd KW20 (for details see Materials and methods). Data represent the mean values of two independent measurements with SD error bars. As determined from the Hanes–Woolf Plot, *K*_M_ amounts to 20.1 μM and the *V*_max_ to 3849 nM/min per OD_490_ = 0.5.

To define l-lactate uptake in wild type, *lctP* and *lctD* mutant strains, ^14^C-l-lactate uptake was measured. As shown in Fig. 3, uptake of ^14^C-l-lactate was determined over time. It can be observed, that in *lctP* knockout mutants only 0.25%, corresponding to 0.05 nmol l-lactate uptake was detectable in strain Rd KW20 *lctP* (Fig. 3A), and less than 0.9% (0.18 nmol) in strain NTHi 2019-R *lctP* at time point 4 min (Fig. 3B). According to NCBI blastp analysis, there is no evidence for a conserved GlcA homologue in *H. influenzae* (data not shown). Additionally in *E. coli* GlcA only shows d-lactate and no l-lactate specificity (Núñez et al., 2001), therefore we have no evidence that background accumulation is due to a GlcA homologue. However, it cannot be ruled out, that the low accumulation level we observe may be due to other mechanisms such as membrane attached l-lactate or generated and attached side products. As further shown in Fig. 3, wild type Rd KW20, *lctP*^+^ complemented strain and 2019-R showed similar substrate uptake reaching saturation between 1 and 2 nmol l-lactate. Testing different substrate concentrations an apparent *K*_M_ value of 20.1 μM and a *V*_max_ of 3.85 μM/(min × OD_490_ 0.5) were determined for strain Rd KW20 by the Hanes-Plot (Hanes, 1932) (Fig. 4). Additionally, it was tested whether an *lctD* knockout mutant has the ability to transport l-lactate. As shown (Fig. 3A), only about one tenth (0.1 nmol) of l-lactate taken up by Rd KW20 was transported into *lctD*::*cat* mutant strain after 4 min. This observation indicates that l-lactate dehydrogenase activity is essentially contributing to the LctP transporter function. Genetic interference of an *lctD*::*cat* insertion on *lctP* transcription is excluded, since both genes are encoded in far distant genomic regions (Fleischmann et al., 1995). Interestingly, a similar effect was observed earlier for the nicotine-ribosyl uptake system PnuC/NadR (Merdanovic et al., 2005). In this study it was shown that substrate flow through a permease was also dependent on downstream activity of a membrane localized NAD synthase enzyme. We therefore suggest that although l-lactate is transported through the lactate permease in an *lctD* background, l-lactate molecules are not released into the cytosol unless the substrate is further processed by the enzyme LctD. This would imply protein/protein interaction between LctP and LctD, which needs further characterization.

### l-Lactate utilization is feedback regulated by its substrate and Woolf the ArcAB system

**Fig. 5:**
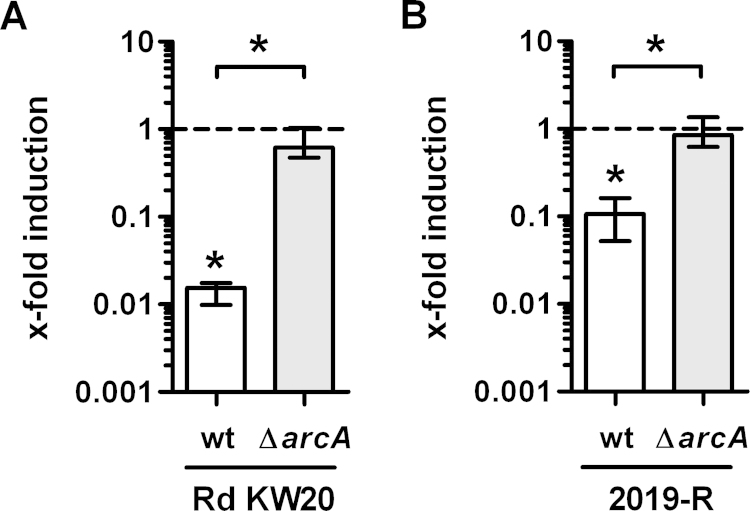
Transcription of *lctP* depends on l-lactate and the ArcAB system. Bars indicate *lctP* mRNA levels of wild type (wt) as well as Δ*arcA* for both, strain Rd KW20 (A) and 2019-R (B). Shown are the *lctP* expression levels of cultures grown in the presence of l-lactate compared to those of cultures grown in the absence of l-lactate (set to 1), both normalized to the housekeeping gene *rpoA*. Each data set represents the median and interquartile range of six independent measurements. Significant differences between the data sets and the control condition as well as between the wild type and the mutant are marked by asterisks (*P* < 0.05; Wilcoxon signed rank test and Mann–Whitney *U* Test, respectively).

In order to determine whether lactate utilization is an inducible system it was characterized if wild type Rd KW20 and wild type 2019-R strains pre-incubated with l-lactate are showing an altered expression and activity of LctP. First, it was found that mRNA levels of *lctP* transcription were different depending on whether bacteria were cultured in Mlc medium with or without l-lactate (7 mM). For Rd KW20 a 65-fold decreased *lctP* transcription was observed in cultures grown with l-lactate (Fig. 5A). By testing NTHI 2019-R strain under the same conditions, it was found that bacteria grown with l-lactate showed an about 10-fold decrease in *lctP* mRNA levels (Fig. 5B). These phenotypes of both wild type strains would not indicate a repressor-regulated system as suggested for the *E. coli* lactate utilization system (Dong et al., 1993), where the presence of lactate induces uptake and l-lactate metabolism. Also, no LctR regulator homologue (Dong et al., 1993) could be found in the annotated genome of *H. influenzae* strain Rd KW20 (Fleischmann et al., 1995). However, as previously published (Wong et al., 2007), *lctP* was identified to be under the control of ArcAB (Georgellis et al., 2001a, Georgellis et al., 2001b). Therefore, it was tested whether an *arcA* knockout mutant strain would still respond to the presence of l-lactate by repressing *lctP* gene expression. As a result it was observed (Fig. 5A, B), that both *arcA* mutant strains no longer showed *lctP* repression in the presence of l-lactate. Additionally, it was observed, as found earlier (Wong et al., 2007), that *lctP* is significantly upregulated in an *arcA* deletion mutant in the Rd KW20 as well as the 2019-R background. A new finding, however, is the observation, whereby l-lactate is a critical substrate and signal molecule for ArcAB activity on the regulation of genes such as *lctP*.

**Fig. 6:**
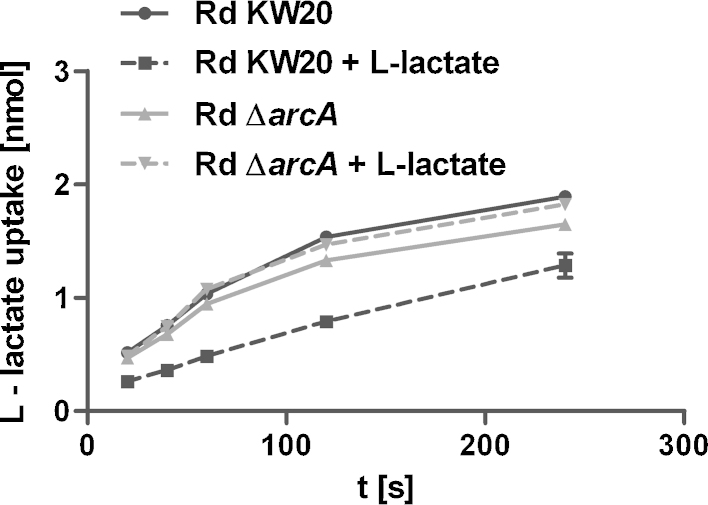
Comparison of l-lactate uptake in Rd KW20 and the *arcA* mutant after growth in Mlc medium with or without l-lactate supplementation. Uptake is measured using l-(3-^14^C)-lactate. Strains are indicated with symbols as presented in the figure. Shown are means and standard deviations of three independent measurements.

To further evaluate an *arcA* effect on l-lactate uptake, ^14^C-l-lactate transport assays were performed. We observed that wild type strain Rd KW20 cultivated without l-lactate showed a higher slope of accumulating ^14^C-l-lactate than does the culture grown in the presence of l-lactate (Fig. 6). Importantly, *arcA* mutants, independently of the presence of l-lactate in the growth medium, show substrate uptake similar to the wild type strain grown without l-lactate. In summary, the qRT-PCR data and the l-lactate transport kinetics conclusively show that *lctP* transcription and substrate uptake activity depend on the ArcAB system. Thus, the ArcAB system is responding to the disposability of l-lactate, leading to alterations of the ArcAB output activity, hence causing a negative feedback regulation on *lctP* transcription.

### Characterization of serum resistance and colonization of NTHi 2019-R

**Fig. 7:**
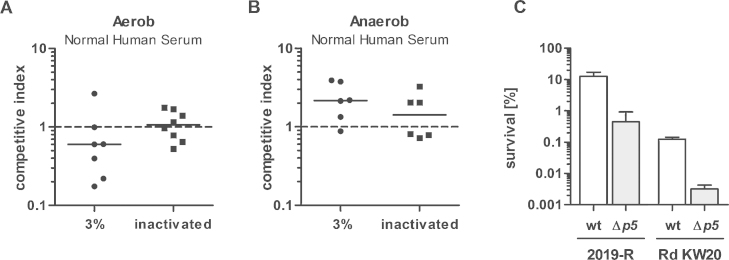
Survival of strain 2019-R versus *lctP* mutant in human blood serum. Shown are the competition indexes, observed after incubation and survival in pooled human serum. Bacteria were grown to mid-exponential phase either aerobically (A) or anaerobically (B). Wild type and mutant strains were mixed in a 1:1 ratio and incubated in 0% (input control), 3% fresh serum and 3% of heat inactivated serum. The competition indexes were determined after plating and counting of wild type versus *lctP* mutant colonies (for details see Materials and methods). Competition index of the 0% serum control was set to 1. As a control (C), Δ*p5* mutants (gray bars) of strain Rd KW20 and 2019-R were compared to the corresponding wild type strains (open bars) for survival in pooled human serum in three independent experiments.

As mentioned in the introduction, for Hib strains it was published that growth in the presence of l-lactate enhances serum resistance (Kuratana et al., 1990, Kuratana and Anderson, 1991). Additionally, in *N. meningitidis*, *lctP* mutants express a significant decrease in serum resistance, further showing that l-lactate stimulates enhanced neuraminic acid production, hence leading to a masking of LOS and finally also to enhanced serum resistance (Exley et al., 2005a). In order to test the clinical NTHi strain 2019-R for l-lactate dependent serum resistance, we compared wild type and the *lctP*::*cat* strain incubated in 3% of pooled human sera, producing a killing of about 95%. These assays were performed by competition analysis. Therefore wild type and *lctP*::*cat* mutant strains were mixed before serum inoculation with either heat inactivated or intact serum. After serum incubation samples were plated and the ratio of wild type to *lctP* mutants (Cm^R^) were determined and indexed. As shown in Fig. 7A, serum killing was unaltered comparing the wild type and the mutant strain after bacteria were grown in the presence of oxygen. Considering a potential role of the ArcAB system, which was previously shown to play a role in serum resistance (De Souza-Hart et al., 2003), we further tested the *lctP* knockout mutant after anaerobic growth. Again, no difference in the survival rates after serum incubation was observed (Fig. 7B). Thus, we conclude that l-lactate is not contributing to serum resistance under the herein tested conditions. Similar experiments were performed with strain Rd KW20 and also no difference was obtained between wild type and Δ*lctP* mutant strain (data not shown). To evaluate the bacteriocidal activity of our human serum, we performed additional serum resistance assays with a *p5* deletion mutant (Fig. 7C), as Rd KW20 Δ*p5* was recently published to be serum sensitive (Rosadini et al., 2013). The results of our assays are in accordance with the published data, showing decreased survival for Rd KW20 and for 2019-R Δ*p5* mutants (Fig. 7C). As these control experiments revealed phenotypes, comparable with the previously published data, we conclude that our serum assay is valid. Therefore we suggest that the deletion of *lctP* and l-lactate utilization does not alter serum resistance.

**Fig. 8:**
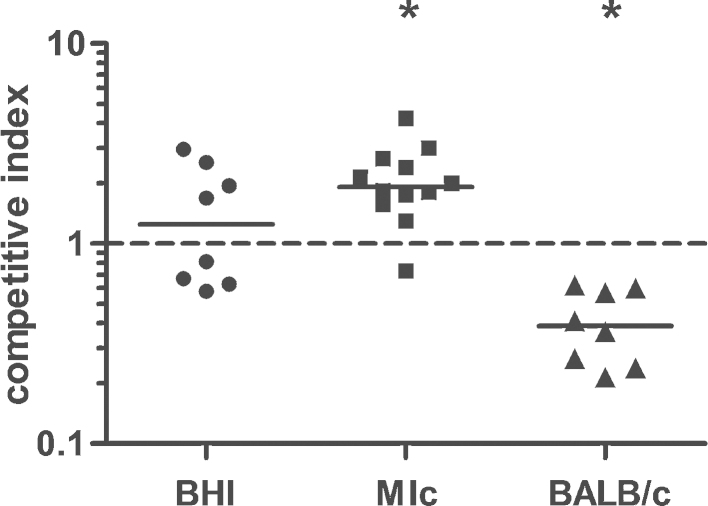
In vitro and in vivo competition of 2019-R and *lctP* mutant. An infection mix, consisting of the wild type and mutant strain in a 1:1 ratio, was applied to each nostril of eight BALB/c mice (in vivo). In parallel, Mlc medium supplemented with l-lactate was inoculated with twelve independent and BHI medium was inoculated with eight independent bacterial suspensions (in vitro). After 24 h the homogenized nasopharynx as well as the inoculated MIc medium were diluted and plated on BHI agar plates. The competitive indexes were calculated after plating and determination of cfu counts (for details see Materials and methods). The asterisks mark a significant difference between the data sets compared to input levels (set to 1) (*P* < 0.0.5, Wilcoxon signed rank test).

To determine an in vivo effect upon l-lactate uptake deficiency, NTHi 2019-R and the corresponding *lctP* mutant strain were tested in a nasopharyngeal colonization assay using 10-weeks old BALB/c mice (Fig. 8). These assays were performed again by competition analysis, using wild type and *lctP*::*cat* mutant strains mixed in a 1:1 ratio before infection. After 24 h nasopharyngeal samples were plated and the ratio of *lctP* mutants (Cm^R^) versus wild type was determined and compared with in vitro competition of the same strains in Mlc and BHI medium. In vitro competition in MIc medium showed an enhanced ratio for *lctP* mutant over the wild type strain (Fig. 8) similar to the faster growth of *lctP* mutant in MIc medium when both wild type strain and *lctP* mutant were grown separately (Fig. 1B, Fig. 2). The competition index determined for cultures grown in BHI indicated same survival of wild type and *lctP* mutant. In contrast, comparison of *lctP* mutant over wild type in the nasopharyngeal colonization assay shows a 2.5 fold decreased colonization ability if compared to input of 1.

In conclusion, l-lactate metabolism is not only important for *Neisseria* sp. (Smith et al., 2007), it was also proposed to affect *H. influenzae* lifestyle (Smith, 2000). However, as l-lactate might give *Neisseria* free direction for host invasion, foremost by increasing serum resistance, no connection could be made between l-lactate and serum resistance for *H. influenzae*. Neither the clinical isolate NTHi 2019-R, nor laboratory strain Rd KW20 (data not shown) express altered serum sensitivity in an *lctP* mutant background. Interestingly and more subtle are the findings in this report, that l-lactate utilization is linked with ArcAB regulation and therefore with possible cell programs of anaerobic redox control. Nasopharyngeal persistence or colonization phenotypes, as determined inhere, might provide a complex picture. l-Lactate could be used as carbon source and may activate ArcAB to fine tune *H. influenzae* metabolisms for its niche, thereby showing impaired colonization of *lctP* mutants. Additionally, and hypothetically l-lactate may influence ArcA controlled LOS biosynthesis genes, such as shown for *lic2B* (Wong et al., 2011) and thereby transiently changes patterns of serum resistance. In the here presented study, we did not observe differences in serum survival between 2019-R and *lctP* mutant, nor for 2019-R incubated with and without l-lactate (data not shown). Furthermore, it was tested whether *lic2B* transcription in strain 2019-R is responding to l-lactate, but no significant alteration of *lic2B* transcription was found (data not shown). Finally *dps* transcription was characterized, which is another trait leading to ArcA regulation (Wong et al., 2007) in strain Rd KW20. However in strain 2019-R *dps* transcription did not respond significantly to l-lactate (data not shown). Thereby, this study shows that strain variation most likely exist for the ArcAB response, futher demonstrated by the different amplitude in *lctP* transcriptional control in both strains tested. Therefore, not only genome wide variations among *H. influenzae* isolates are important to be highlighted, but also different sensory and response activities. The ArcAB system may contribute to these diversifications of phenotypes in distinct strain backgrounds. Finally, the characterization of the l-lactate utilization pathway in *H. influenzae* on the one hand reveals that no clear correlation with serum resistance exists. On the other hand decreased in vivo surviving fitness is observable for NTHi strain 2019-R and as published earlier for the invasive growth of strain Rd-b^+^ (Herbert et al., 2002). Thus, we conclude that l-lactate seems to play a crucial role in *H. influenzae* metabolism and supposedly in global regulation patterns via ArcAB.
